# Length-dependent RNA foci formation and Repeat Associated non-AUG dependent translation in a *C. elegans* G _4_ C _2_ model

**DOI:** 10.17912/micropub.biology.000600

**Published:** 2022-07-15

**Authors:** Todd Lamitina

**Affiliations:** 1 Departments of Pediatrics and Cell Biology, University of Pittsburgh School of Medicine, Pittsburgh, PA, USA

## Abstract

GC-rich repeat expansion mutations are implicated in several neurodegenerative diseases and can lead to repeat associated non-AUG-dependent (RAN) translation and concentrations of nuclear RNA foci. To model *C9orf72* ALS/FTD, we engineered *C. elegans* to express pure GGGGCC (G _4_ C _2_ ) repeats of varying lengths and observed RAN translation and nuclear RNA foci. RNA foci were observed in animals expressing ≥20 G _4_ C _2_ repeats while RAN translation occured in animals expressing ≥33 G _4_ C _2_ repeats. These findings show that in *C. elegans* , RAN translation can occur even in the absence of *C9orf72* intronic sequence normally surrounding the repeat. Given that the currently accepted repeat threshold for C9 disease is >30 repeats, our data are consistent with a model in which RAN peptides are key drivers of *C9orf72* disease pathology.

**Figure 1.**
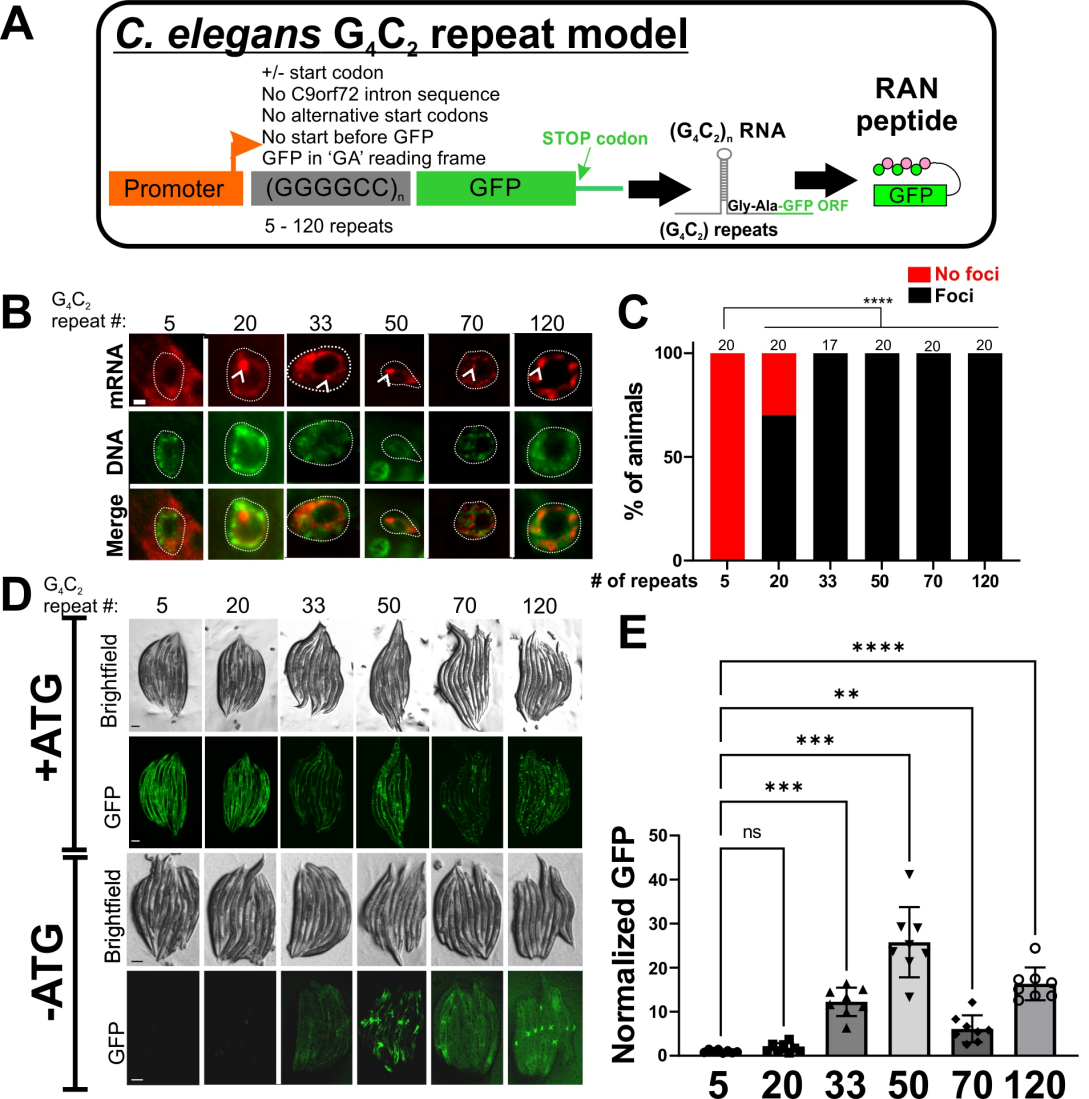
**
Figure 1. Characterization of a *C. elegans* G _4_ C _2_ repeat model** . A) Description of G _4_ C _2_ expression clones and their expression products. Characteristics of these expression clones are listed above the G _4_ C _2_ repeat domain. B) *In situ* hybridization to detect G _4_ C _2_ -containing mRNA in muscle cell nuclei. Arrowheads point to examples of RNA foci, which were only observed in muscle cell nuclei (dashed circle). Scale bar = 1 micron. C) Quantification of the percentage of animals with observable RNA foci. The number above each bar represents the number of animals examined. **** - p<0.0001, Fisher’s exact test with Bonferroni correction. D) Canonical translation or RAN translation was detected using GFP expression. Note in the ‘+ATG’ lines how GFP expression transitions from soluble to puncta as the number of repeats increase. This is consistent with the production of a (Gly-Ala)x-GFP fusion protein with increasing number of repeats, which is known to form aggregates in *C. elegans* (Rudich *et al.* 2017). Without a start ATG, GFP expression is not observed until repeats ≥33, which fits the definition of RAN translation (Zu *et al.* 2011). All images were taken using identical exposure times. Scale bar = 100 microns. E) Quantification of RAN translated GFP. Data were normalized to the mean of the 5 repeat measurements. Graph shows the mean ± S.D. with individual data points shown. **-p<0.01, ***-p<0.001, ****-p<0.0001, One-way ANOVA with Welch test.

## Description

A six nucleotide repeat (GGGGCC; G _4_ C _2_ ) in the first intron of the *C9orf72* gene is the most common genetic cause of Amyotrophic Sclerosis (ALS) and Frontotemporal Dementia (FTD) (DeJesus-Hernandez *et al.* 2011; Murray *et al.* 2011; Renton *et al.* 2011). The formation of nuclear localized repeat-containing RNA foci is a hallmark of *C9orf72* pathogenesis (Mizielinska *et al.* 2013). Moreover, unconventional repeat associated non-AUG dependent (RAN) translation of sense G _4_ C _2_ and antisense G _2_ C _4_ repeat containing mRNA gives rise to six distinct dipeptide proteins (DPRs) (Mori *et al.* 2013), some of which are highly neurotoxic in multiple model systems, including *C. elegans* (Rudich *et al.* 2017; Snoznik *et al.* 2021). While prior studies have shown G _4_ C _2_ repeats can give rise to RNA foci and RAN translation in *C. elegans* (Kramer *et al.* 2016; Sonobe *et al.* 2021), the contextual requirements for these processes are not defined. For example, how many G _4_ C _2_ repeats are required for RNA foci formation and RAN translation? Is the sequence surrounding the G _4_ C _2_ repeat in intron 1 of the C9orf72 sequence required for RAN translation?

To address these questions, we developed transgenic *C. elegans* expressing defined numbers of pure G _4_ C _2_ repeats lacking any of the surrounding *C9orf72* intron sequence (Figure 1A). To clone these 100% GC repeats, we commercially synthesized a (G _4_ C _2_ ) _5_ clone flanked by unique restriction sites (HindIII/BamHI) and expanded the repeat length using a recursive directional ligation strategy (McDaniel *et al.* 2010) into the standard *C. elegans* pPD95.79 GFP expression vector. To achieve repeat sizes ≥33, we discovered that we had to modify the pPD95.79 vector by reversing the origin of replication (Ori), likely due to alterations in the stability of G-rich leading versus lagging strands during replication (Thys and Wang 2015). With the Ori flipped, we were able to clone up to 120 G _4_ C _2_ repeats, which we verified by restriction digest and DNA sequencing. We cloned a muscle-specific *myo-3* promoter (with or without a start ATG) upstream of the repeat. GFP coding sequence was downstream of the repeat and was located in the ‘Glycine-Alanine’ (GA) DPR reading frame. We used these plasmids to generate transgene arrays using standard microinjection approaches. Repeats *in vivo* appeared stable, since in the ‘+ATG’ clones, we observed robust GFP expression that showed increased aggregate formation with increasing repeat number, consistent with our previous observations of GA-GFP (Rudich *et al.* 2017). We did not observe notable changes in viability, fecundity, or behavior for any of the lines. We also failed to observe any notable phenotypes when the 120 repeat clone was injected at higher concentrations (100ng/µl). Note that unlike previous *C. elegans* G _4_ C _2_ models (Kramer *et al.* 2016; Sonobe *et al.* 2021), our models lack any neighboring *C9orf72* sequence context.

We first examined the properties of RNA foci formation in Day 1 adults using *in situ* hybridization to detect sense strand RNA foci (Figure 1B,C). We observed that expression of high repeat numbers gave rise to robust nuclear foci only in the muscle cells expressing the G _4_ C _2_ mRNA. We failed to detect RNA foci in muscle using a G _4_ C _2_ antisense probe, suggesting these *C. elegans* G _4_ C _2_ lines do not undergo antisense transcription as is observed with native G _4_ C _2_ repeats in mammals (Zu *et al.* 2013). We discovered that the threshold for sense RNA foci formation was less than 20 repeats, as no foci formation was observed in the 5 repeat animals, but foci were observed in 20, 33, 50, 70, and 120 repeat animals. Qualitatively, RNA foci appear to be more discreet and numerous as the number of repeats increase, although we note that this could be a consequence of the number of available binding sites for the nucleic acid probe, which is 4 repeats in length.

Next, we examined whether or not the G _4_ C _2_ transgenes lacking a start ATG supported RAN translation, despite the absence of surrounding *C9orf72* intron sequence (Figure 1D,E). All of the G _4_ C _2_ clones produced robust GFP expression in the presence of a start ATG, indicating all of the clones are intact and capable of supporting translation. However, in the absence of a start ATG, we did not observe GFP expression in lines with 5 or 20 G _4_ C _2_ repeats. However, in animals expressing 33, 50, 70, and 120 G _4_ C _2_ repeats, significant GFP expression in muscle cells was observed. The levels of GFP expression in the -ATG lines undergoing RAN translation were qualitatively weaker that those observed in the +ATG lines. Additionally, we found that GFP expression in the -ATG lines rarely formed puncta as observed in the +ATG lines with ≥33 repeats. This could be due to the lower levels of GFP expression in the -ATG lines, since aggregate formation may be concentration dependent (Fung *et al.* 2003). Alternatively, this could be due to the initiation of translation within the body of the repeat leading to the production of GA repeats below the threshold for aggregation. Nevertheless, our data show that *C. elegans* exhibit repeat-associated non-ATG dependent translation from a pure G _4_ C _2_ repeat transgene lacking any additional *C9orf72* -specific sequences.

We find that the pure G _4_ C _2_ sequence is sufficient to support RNA foci formation and RAN translation even in the absence of any flanking *C9orf72* sequences in *C. elegans* . This is significant because all previous *C. elegans* G _4_ C _2_ models utilized a *C9orf72* ‘minigene’ containing a single repeat length with flanking human intronic sequence. These intronic sequences may play an important role since RAN translation is greatly enhanced by the presence of non-repeat alternative start codons within the *C9orf72* intron (Green *et al.* 2017; Sonobe *et al.* 2021). Our findings show that even in the absence of these intronic sequences, the pure repeat sequence is sufficient to support both RNA foci formation and RAN translation.

G _4_ C _2_ RAN thresholds in *C. elegans* closely match the hypothesized disease threshold in human ALS patients of >30 repeats (DeJesus-Hernandez *et al.* 2011). This is consistent with the hypothesis that RAN peptides have a primary pathological role in the development of C9 disease. Indeed, we previously found that arginine-rich dipeptide repeats are toxic in both *C. elegans* and mammals through similar genetic pathways (Snoznik *et al.* 2021). Overall, our G _4_ C _2_ models provides a robust visual phenotype for future genetic screens aimed at defining the molecular mechanisms of G _4_ C _2_ RAN translation. Such screens may provide new pathological insights and treatments targets for the *C9orf72* related neurodegenerative diseases.

## Methods

**Construction of G _4_ C _2_ expression plasmids and transgenic worms** . A (GGGGCC) _5_ sequence flanked by HindIII and BamHI restriction sites was commercially synthesized in the pMA vector (ThermoFisher Scientific). Repeats were expanded using the recursive directional ligation (RDL) strategy to generate pure uninterrupted repeats, as previously described (Mizielinska et al. 2014). Briefly, the origin vector was linearized with BspQI. An insert of (G _4_ C _2_ ) _n_ was isolated via BspQI/EcoO1091 digestion and inserted into the origin vector. Further rounds of RDL were carried out to generate repeats of the indicated sizes. Repeats were subcloned into the pPD95.79 vector (AddGene) using HindIII/BamHI digestion. This vector was modified to reverse the origin of replication, which we discovered to be necessary to subclone >33 G _4_ C _2_ repeats. A 2.5Kb *myo-3* promoter fragment +/- the start ATG was subcloned into the HindIII site to generate the final expression vector. Final expression clones were verified by Sanger sequencing (Genewiz). Chromatographs were visually inspected to verify the number of GGGGCC repeats. N2 worms were injected with the G _4_ C _2_ expression plasmid (20 ng/µl) and either *rol-6* (80 ng/µl) or *myo-2p::mCherry* (2.5 ng/µl) as a transformation marker. At least 2 extrachromosomal array lines were analyzed for each repeat construct to verify phenotypic consistency. All strains were grown on NGM plates with OP50 bacteria.

***In situ* hybridization** . RNA foci were detected using *in situ* hybridization using a Cy5-labeled (G _2_ C _4_ ) _4_ locked nucleic acid (LNA) probe (IDT). Day 1 adult worms grown at 25ºC were fixed in 4% paraformaldehyde followed by washes in 70%, 90%, and 100% EtOH. The LNA probe was denatured at 80 ºC for 75 seconds and then added to hybridization buffer (50% formamide, 2X SSC, 50 mM NaPO4, 10% dextran sulfate) at 10 ng/µl. Probes were hybridized overnight at 37 ºC. Worms were then washed twice for 30 minutes with 2X SSC at 37 ºC. Next, worms were washed in 4X SSC with 0.1% Triton X100 containing 1 ug/ml Hoechst 33258, followed by two washes with 2X SSC at room temperature for 5 minutes. Worms were then mounted on slides containing antifade solution. Images were acquired using a Leica DMI4000B widefield microscope and deconvolved using identical settings. Each image is from a deconvolved Z-stack where DNA and RNA foci were observed in the same plane. For quantification, individual animals were scored as either foci positive or foci negative by an observer blinded to genotype. Animals was scored as ‘Foci positive’ if they contained ≥5 foci of Cy5 signal that co-localized with the nuclear DNA signal.

**GFP imaging** . Worms expressing the indicated number of G _4_ C _2_ repeats were cultured at 25 ºC. 24 hours after worms reached the L4 stage, animals were anesthetized with 10 mM levamisole. For images, worms were arranged on the surface of a clean NGM agar plate and images were captured using M205FA fluorescence dissecting scope and a DFC345FX digital camera using identical exposure times within the '+ATG' or '-ATG' groups. For quantification, worms were anesthetized in 10mM levamisole and placed on a slide and GFP and DAPI images were captured with a 10X lens and GFP filter (Leica L5 ET set) and DAPI filter (Leica A4 ET set) on a DMI4000B with a DFC 340FX camera (N=8 per genotype, identical settings for all genotypes). To account for autofluorescence, the DAPI image was subtracted from the GFP image. For the resulting subtracted image, GFP fluorescence was quantified along each of three 100 micron lines per animal centered on the midline at the animal’s head (immediately posterior to pharynx), midbody (vulva region), and tail (immediately posterior to intestine). Intensity measurements were summed along and between lines to get a total GFP measurement per animal). Image subtraction and intensity measurements were carried out in Leica Advanced Fluorescence Software, v2.1.0.

## Reagents

*C. elegans* Strains:

**Table:** 

**Strain**	**Genotype**
OG1247	+/+; *drEx503 [myo-3p+ATG::(G _4_ C _2_ ) _5_ -gfp (20ng/ul); rol-6 (80ng/ul)* ]
OG1249	+/+; *drEx505[myo-3p+ATG::(G _4_ C _2_ ) _20_ -gfp (20ng/ul); rol-6 (80ng/ul)* ]
OG756	+/+; *drEx275 [myo-3p+ATG::(G _4_ C _2_ ) _33_ -gfp (20ng/ul); rol-6 (80ng/ul)] *
OG794	+/+; *drEx301 [myo-3p+ATG::(G _4_ C _2_ ) _50_ -gfp (20ng/ul); rol-6(80ng/ul)] *
OG779	* oxIs322; drEx287 [myo-3p+ATG::(G _4_ C _2_ ) _70_ -gfp (20ng/ul); rol-6 (80ng/ul)] *
OG791	* oxIs322; drEx298 [myo-3p+ATG::(G _4_ C _2_ ) _120_ -gfp (20ng/ul); rol-6 (80ng/ul) *
OG1148	* +/+; drEx480 [myo-3p-NO ATG::(G _4_ C _2_ ) _5_ -gfp (20ng/ul); rol-6 (80ng/ul)] *
OG1145	* +/+; drEx477 [myo-3p-NO ATG::(G _4_ C _2_ ) _20_ -gfp (20ng/ul); rol-6 (80ng/ul)] *
OG759	* +/+; drEx477 [myo-3p-NO ATG::(G _4_ C _2_ ) _33_ -gfp (20ng/ul); rol-6 (80ng/ul)] *
OG967	* drIs42 [myo-3p-NO ATG::(G _4_ C _2_ ) _50_ -gfp (20ng/ul); rol-6 (80ng/ul)] *
OG782	* oxIs322; drEx290 [myo-3p-NO ATG::(G _4_ C _2_ ) _70_ -gfp (20ng/ul); rol-6 (80ng/ul)] *
OG977	* drIs41 [myo-3p-NO ATG::(G _4_ C _2_ ) _120_ -gfp (20ng/ul); myo-2p::mCherry (2.5ng/ul); pBluescript (77.5ng/ul)] *
